# Does Market Performance (Tobin’s Q) Have A Negative Effect On Credit Ratings? Evidence From South Korea

**DOI:** 10.1007/s10690-023-09406-x

**Published:** 2023-05-10

**Authors:** Hyoung-Joo Lim, Dafydd Mali

**Affiliations:** 1 Kyonggi University, 154-42 Kwangkyo San-Ro, Youngtong Gu, Suwon, Seoul, Gyounggi South Korea; 2 University of Nottingham, Jubilee Campus, 7301 Wollaton Rd, Lenton, Nottingham, NG8 1BB England, UK

**Keywords:** Credit ratings, Tobin’s Q, Accounting education, Mispricing/overvaluation, M40, M41, M15, M21

## Abstract

Tobin’s Q is an established measure of firm performance, based on investor confidence. However, the association between Tobin’s Q and credit ratings is not well-established in the literature. Using a sample of Korean listed firms over the 2001–2016 sample period, Probit regression analysis shows that overall, Tobin’s Q is positively associated with credit ratings. However, for firms with a > 1 (1 <) Tobin’s Q ratio, a negative (positive) relationship exists. Moreover, in independent regressions, a threshold level if found where the effect of Tobin’s Q on credit ratings changes from being positive (0.2), to negative (0.3). To the best of our knowledge, we are the first to demonstrate that credit rating agencies are nuanced when making default risk assessments. Specifically, that in South Korea, a threshold level exists, at which increasing Tobin’s Q values reduce credit ratings. Empirical evidence of the different association between Tobin’s Q (market confidence) and credit ratings can extend the literature and offer insights to market participants. Furthermore, because Tobin’s Q is a commonly used proxy for financial performance in accounting lectures, the study has practical implications for academics in classrooms.

## Introduction

Rating agencies issue credit ratings as an estimate of a firm's ability to survive a business cycle. A firm's credit rating can therefore be considered by market participants as a meaningful and economically significant estimator for a firm's default risk. The extant literature shows that credit ratings are influenced by a firm’s financial indicators (Hovakimian et al., 2009; Alissa et al., 2013; Kraft, 2014; Ashbaugh-Skaife et al., 2006; Ziebart and Teiter, 1992; Kaplan & Urwitz, 1979). In addition to financial data, ‘soft’ data is impounded into credit ratings based on governance data (Bhojraj & Sengupta, 2003; Ashbaugh-Skaife et al., 2006; Crouchy et al., 2001). In the extant literature, firm value (overall market performance) is captured using Tobin’s Q (Q ratio). In countless studies, Tobin’s Q replaces return on asset (ROA) and other simple accounting ratios, to proxy financial performance. Thus, it may be expected that Tobin’s Q will have a positive effect on credit ratings. However, surprisingly, the association between Tobin’s Q and credit rating is not well-established in the literature.

Tobin’s Q is estimated as a firm’s total equity value, divided by the book value of assets. The Q ratio is an accepted measure of investor confidence (Farrell & Gallagher, 2015; Gatzert & Schubert, 2022; Hwang & Kim, 2018; Upadhyay, 2015). For example, a firm with a Tobin’s Q of 0.4 has more assets listed on financial statements compared to its equity market value. In such a situation, a firm's equity is of a lower value to its assets, due to low market confidence. As Tobin’s Q increases to a level of 1, equity increases relative to assets, implying higher market/investor confidence. The study’s main research question is: *Does market performance (Tobin’s Q) have a negative effect on credit ratings?* We envision that a relationship exists. However, credit rating agencies may be nuanced in their credit rating status allocation, based on the following assertion. (I) Based on a Tobin’s Q value of above 1, a firm can be considered as demonstrating strong market performance. This firm also has enough assets to cover equity requirements. (II) On the other hand, a firm with a higher Tobin’s Q of 2 demonstrates higher market performance. But this firm does not have enough assets to cover its equity. Thus, from the perspective of a credit rating agency, in an instance of potential mispricing or overvaluation, the firm with a Tobin’s Q value of 2 has a stronger potential to default, despite investor confidence. Whilst the aforementioned different associations are likely to exist, it is not previously reported in the extant literature. Thus, the purpose of this study is to add granularity, by empirically demonstrating whether credit rating agencies are nuanced in their credit rating assessment, based on Tobin’s Q.

Using a sample of Korean firms over the 2001–2016 sample period, Tobin’s Q is shown to have a positive association with credit ratings. However, when the sample is divided into firms with a Q ratio of below and above 1, the relationship for firms with a 1 < Tobin’s Q remains positive. However, a negative relationship exists between credit ratings and Tobin’s Q for the > 1 Tobin’s Q sample. Upon further investigation, a positive association between Tobin’s Q and credit ratings is captured in regressions for the 0–0.2 Tobin’s Q sample. However, above 0.3, the result is negative. We observe qualitatively similar results after partitioning the sample into Investment Grade (IG)/Non-Investment Grade (NIG) samples. To add robustness to the model, we control for important endogenous variables, (1) ratings in previous year (2) rating changes, (3) fixed firm and year effects, and (4) credit ratings in period t + 1 and t + 2. Empirical results are consistent for all additional analyses.

The findings make important contributions and enhance the literature as follows. First, it is accepted that firm performance indicators such as, profitability, leverage, size, liquidity, corporate governance, amongst others are impounded into credit ratings, as indicators of default risk. Therefore, a positive relationship can be expected between credit ratings and Tobin’s Q. In the initial analysis, we find that Tobin’s Q has a positive effect on credit ratings, using the full sample. However, when the sample is divided into Q ratios of > 1/1 < , the results imply that credit rating agencies interpret the relationship between Tobin’s Q and credit ratings differently for both groups. The negative relationship between Tobin’s Q and credit ratings suggest that firms with a Tobin’s Q of > 1 are less likely to survive the business cycle as Tobin’s Q increases, compared to firms with Tobin’s Q values of 1 < . The results may be interpreted as follows. Firms with Tobin’s Q values of 1 (equity): 1 (assets) are able to repay investors and shareholders if required with less difficulty compared to firms with a Tobin’s Q value of 2 (equity): 1 (assets). Thus, despite investor confidence, firms with higher equity to assets may be perceived as more likely to default by credit ratings analysts, compared to firms with higher equity values, due to the potential to mispricing or overvaluation.

Second, after showing that a different relationship exists between the 1 < / > 1 samples, we demonstrate that a point exists at which the positive relationship between Q ratios and credit ratings become negative. Overall, for firms with a Tobin’s Q value of 0–0.2, a positive relationship exists between Tobin’s Q and credit ratings. However, as firms Q ratios increase above 0.3, the relationship between Q ratios and credit ratings become negative. The result can be interpreted as follows. For the firms with the lowest level of market confidence, increasing market performance has a strong positive effect on credit ratings. For example, a firm with a market value of 0.05 can increase its value by 400% to have a market value of 0.2. The results therefore infer that credit rating agencies are nuanced in their default risk assessments, based on firms with lower levels of Tobin’s Q.

Third, South Korea is a unique geographical location to conduct this study. Legislative oversight was a major contributing factor in the 1997 Financial Crisis (LaPorta, 1997; Emst, 1998). To enhance financial reporting transparency, South Korea is shown to be an early adopter of regulation policy (Choi et al., 2018; Lim & Mali, 2020, 2022, 2023; Mali & Lim, 2018, 2019, 2020). In 2023, South Korea is now the 10th largest economy based on GDP. Therefore, this study provides a unique opportunity for international comparative analysis, to demonstrate whether the aforementioned credit risk/Tobin’s Q relationship is commonplace, or whether it is a result of South Korea’s unique situation. Finally, as discussed in the conclusion, ROA and Tobin’s Q are discussed in the same breath in accounting lectures. The study can therefore inform academics about how to introduce Tobin’s Q in terms of risk and reward.

The paper proceeds as follows. In Sect. 2, relevant literature is introduced, and hypotheses are developed. Section 3 provides details about data collection and research design. In Sect. 4, the results of empirical results are discussed. Section 5 includes additional robustness tests. Section 6 concludes.

## Literature Review

### Previous Literature

The primary role of a credit rating agency is to provide market participants with an indication of whether a firm is likely to survive through a business cycle (Carey & Hrycay, 2001). A firm’s credit rating is used by market participants as an estimate of a firm’s default risk potential, with credit ratings providing an economically meaningful measurement of risk for investment purposes (Boot et al., 2006). Firms with a similar credit rating are grouped together as firms of similar quality (Kisgen, 2006). In South Korea, the largest credit ratings agency, NICE (National Information & Credit Evaluation) provides firms with 10 broad credit ratings. A value of 10 (1) is estimated as the lowest (highest) level of default risk. Credit ratings are important to management because firms with lower credit ratings are disadvantaged in terms of reputation, borrowing costs, rates and reduced terms from suppliers (Blume et al., 1998; Dichev & Piotroski, 2001; Ederington & Goh, 1998; Holthausen & Leftwich, 1986). The literature suggests that whilst management have an incentive to enhance credit ratings through managerial interventions, they are not successful (Ali & Zhang, 2008; Alissa et al., 2013; Hovakimian et al., 2001, 2009; Jung et al., 2013; Kisgen, 2006). Taken together, the literature suggests that market participants can consider credit ratings as both an important and independent measurement of a firm’s default risk.

Credit ratings are estimated by agencies using a combination of internal (hard) financial firm data and ‘soft’ data. Previous empirical studies show that financial statement data including earnings, competitive position, size, stability, profitability, leverage, and financial strength influence a firm’s credit ratings (Hovakimian et al., 2009; Alissa et al., 2013; Kraft, 2014; Ashbaugh-Skaife et al., 2006; Ziebart and Teiter, 1992; Kaplan & Urwitz, 1979). In addition to financial statement data, credit rating analysts make adjustments based on ‘soft’ data that includes managerial credibility (Kraft, 2014) and corporate governance (Bhojraj & Sengupta, 2003; Ashbaugh-Skaife et al., 2006; Crouchy et al., 2001). Market performance is also shown to influences a firm’s ability to survive the business cycle (Lim and Mali, 2019). Taken together, the literature provides evidence that firm performance is positively associated with credit rating status. Therefore, it is surprising that the association between credit ratings and Tobin’s Q is not widely reported in the literature.

AI reasoning systems and machine learning techniques are the established tools to estimate credit risk by rating agencies (Galindo et al., 2000). Huang et al. (2004) surmises that structures developed by humans are relatively simple and easy to interpret, while AI models and machine learning methods are usually very complicated and difficult to explain. AI machine learning methods are considered a well suited and efficient estimation of credit ratings (Kwon et al., 1997; Shin et al., 2001). However, Shin (2001) suggests that the drawback of AI and machine learning is that metrics may be difficult to decipher. To interpret the models developed by credit rating agencies, researchers use regression analysis to capture the ‘average’ effect of firm performance on credit ratings. However, there is the potential that the relationship between Tobin’s Q and credit ratings may be complex. In this study, we interpret that credit rating agencies can impound Tobin’s Q into credit rating assessment, based on two assertions; (i) a positive association, based on market performance, (ii) or a situation in which firms do not have the ability to repay shareholders, based on an overvaluation or mispricing assertion (negative).

James Tobin’s initial model developed in 1969 was designed to capture a firm’s intrinsic value. The model has been developed by Lucas and Prescott (1971), Abel (1983) and the model that is predominantly used in academic studies is a Tobin’s Q model developed by Hayashi (1982). Hayashi’s (1982) Q ratio measures a firm’s average replacement cost of assets relative to the market cost of asset based on perfect competition. In 2023, Tobin’s Q, market performance assertions are regularly taught at undergraduate level as a proxy of future firm performance, based on investor confidence. Based on the commonly held belief in extant literature, a firm with a Tobin’s Q of 0.4 can be considered undervalued as a result of weak investor sentiment. A firm with a Tobin’s Q above 1 can be considered as demonstrating higher market value relative to assets. A firm with a Tobin’s Q of 2 is framed as having higher market value, relative to assets, due to higher investor confidence.

Various studies suggest Tobin’s Q has a positive relationship with firm performance (Berger & Ofek, 1995; Khan et al., Sarhan et al., 2019; Bi & Wang, 2018). There is also evidence that governance and management effort is associated with Tobin’s Q (Abdullah et al., 2015; Al‐Najjar, 2012; Elsayed & Elbardan, 2018). However, Dybvig and Warachka (2015) suggest that Tobin’s Q may distort true firm performance because management may reduce assets relative to stock to increase Tobin’s Q. Moreover, Philippon, (2009) demonstrates that equity has a higher likelihood of being mispriced compared to debt. Taken together, whilst increasing Tobin’s Q can signal increased market performance, it can also lead to opaqueness because higher (lower) market value (assets) reduces a firm's ability to repay shareholders. Therefore, potentially, academic tension exists in the literature regarding the association between credit ratings and Tobin’s Q, and whether (at what point does) market confidence becomes an overvaluation, and reduces a firm’s credit ratings, due to mispricing.

### Hypothesis Development

**Fig. 1:**
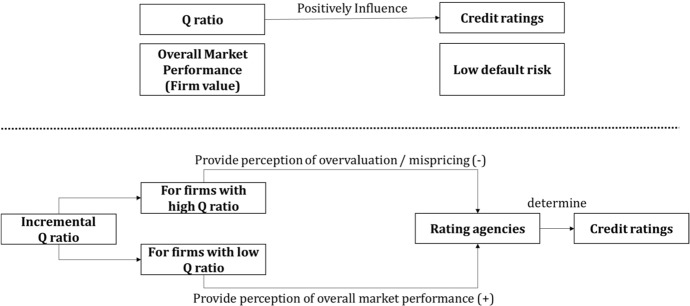
The relationship between Q ratio and credit ratings

Tobin’s Q and relative stock prices vary due to investment decision making, which can be contextualized based on the efficient market hypothesis (EMH). EMH suggests investors are profit maximizing and utilize all valuable information for investment purposes. If market participants believe that a firm has the potential for upside opportunity, market confidence will increase stock price relative to assets. For example, a firm with a Tobin’s Q of 0.75 compared to 0.1 can be considered as demonstrating the potential for higher market returns based on investor confidence. This relationship can be interpreted by credit rating analysts and captured as a positive relation between Tobin’s Q and credit ratings based on analyst’s belief that a firm with higher market confidence are more likely to survive the business cycle compared to a firm with a lower Tobin’s Q (market performance). Overall, we believe that the association between Tobin’s Q and credit ratings will be positive, for a sample of Korean listed firms, as illustrated in Fig. 1. Based on the above, we develop the following hypothesis:

#### H1

Tobin’s Q is likely to have a positive effect on credit ratings.

However, the accounting equation is defined as Assets = Liabilities + Owner’s Equity. In a situation where the book value of assets (1) are at parity with a firm’s equity (1), a firm with a Tobin’s Q of 1 can be considered as having sufficient assets to repay all debt and equity commitments. However, as Tobin’s Q increases to > 1, the firm will become increasingly less likely to reimburse investors for their equity share, compared to firms with Tobin’s Q levels of 1 < . Thus, in situations of robust market performance, credit ratings analysts may interpret that higher Tobin’s Q levels increases potential default risk, based on potential overvaluation or mispricing. On the other hand, a firm with a Tobin’s Q of 1 < can be considered more likely to survive the business cycle because the firm will have the ability to repay investors if investors have an incentive to sell their equity investment.

Above, we hypothesize that firms with Tobin’s Q levels of 1 < and > 1 may have different levels of risk from a credit rating analyst's perception. However, whilst we hypothesize that the relationship between credit ratings and Q ratio can be positive (negative) for the < 1 (> 1) group, we also surmise that a point is likely to exists at which market confidence become an overvaluation and reduces a firm’s credit ratings. At this point, after controlling for firm risk determinants, credit ratings will consider that a firm is less likely to survive a business cycle, thus, allocate a lower credit rating. Whilst this 'threshold' Tobin’s Q level has not been reported in the literature, we posit that such a relationship is likely to exist and can be discovered using empirical testing, due to the fact that credit rating analysts have various AI and machine learning systems at their disposal. In the first hypothesis, we believe that overall, a positive relation is likely to exist between credit ratings and Tobin’s Q. However, in the second hypothesis, we assert that firms that are overvalued (> 1, or at a 'threshold' level) with relatively less assets to repay investor are also likely to demonstrate high levels of default risk. Based on the above, we develop the following hypothesis:

#### H2

For firms with high Tobin’s Q values, lower credit ratings will be imparted by rating agencies.

## Research Design

Tobin’s Q is estimated in Eq. (1). The value is acknowledged by the Korean Stock Exchange (KRX), as an accepted measurement of market performance. Credit ratings are estimated as the ordinal rank of credit ratings. In Table 1, we provide details of how credit rating ordinal rankings are equivalent to Moody’s and S&P. Firms with the highest credit rating value of 10, represent firms with a credit rating of AAA. Firms with a credit rating value of 1, represent firms with a credit rating of C for Moody’s and D for S&P. This methodology is established in previous South Korean studies (Lim & Mali, 2018; Mali & Lim, 2019). As explained in the first hypothesis, we expect to find a positive relationship between Tobin’s Q and credit ratings for our full sample. However, in situations of high Tobin’s Q, because of the potential for overvaluation/mispricing, a negative relation between Tobin’s Q and credit ratings has the potential to exist (second hypothesis).

1 $$TQ=(MVCS+MVPS+STL-STA+LTD-INV)/TA$$

where

**Table 1:** Credit ratings coding

CR	IG/NIG	Grade	Definition	Moody's	S&P
10	IG	Best grade	Extremely strong	Aaa	AAA
9		High grade	Very strong	Aa1 & Aa2	AA + & AA
8			Strong	Aa3	AA-
7		Middle grade	Good	A1 & A2	A + & A
6			Medium	A3	A-
5			Less vulnerable	Baa1 & Baa2	BBB + & BBB
4	NIG	Low grade	More vulnerable	Baa3	BBB-
3		Poor grade	Currently vulnerable	Ba & B &Caa	B & C & CCC
2			Highly vulnerable	Ca	C
1			Extremely vulnerable	C	D

*TQ*: Tobin’s Q, *MVCS*: Market value of common stock, *MVPS*: Market value of preferred stock, *STL*: Short term (current) liabilities, *STA*: Short term (current) assets, *LTL*: Long term debt, *INV*: Inventory, *TA*: Book Value of Total assets.

In Table 2, control variables are divided into established credit ratings determinants; business risk, firm size, liquidity, firm performance and ownership (governance) structures. The study adopts Woodside’s (2016) suggestion that only including determinants that influence the dependent variable enhances the predictive validity of empirical tests. Woodside (2016) infers that whilst there is a trend to include statistically insignificant variables in modern studies, this approach reduces the quality of empirical findings. Thus, prior to the analysis, a battery of tests have been conducted to establish the most appropriate variable for each determinant.

**Table 2:** Variable definitions and Sample selection

Variable	Sign	Category	Definition	Variable	Sign	Category	Definition
*Panel A: variable definitions*							
*Credit rating*	+ /–	Variable of interest	Credit ratings, ordinal rank 1 to 10	*Size*	+	Firm size	Natural logarithm of market capitalization
*TobinQ*	+	Market performance	Tobin’s Q calculated using Chung and Pruitt (1994)	*Current_R*	+	Short-term liquidity	Current assets/Current liabilities
*Lev*	–	Business risk	Debt ratio (= Total liabilities/Total assets)	*ROA*	+	Firm Performance	Return on assets (= Net income/Total assets)
*SBTB*	–	Business risk	Short term borrowings/Total borrowings	*BigOwn*	+	Governance structure	Biggest shareholder’s share holdings(%)
*Borrowing*	–	Business risk	Total borrowings/Total liabilities	*Foreign*	+	Governance structure	Foreign investors’ share holdings(%)
*ABDA*	–	Business risk	Absolute value of discretionary accruals suggested by Dechow et al. (1995)	*ID*		Fixed effect	Industry fixed effect
*TRM*	–	Business risk	Total amount of real earnings management suggested by Cohen and Zarowin (2010)	*YD*		Fixed effect	Year fixed effect
Panel B: Sample selection				*YD*		Fixed effect	Year fixed effect
Initial sample							14,733
Excluding firms with no audit hour/financial data available							(1642)
Final sample							13,091

We control for business risk using firm leverage, short term borrowing, total borrowing, accrual earnings management and real earnings management. A negative relationship is expected between leverage and credit ratings because firms with higher levels of debt to equity are less likely to survive the business cycle compared to firms with lower debt. Based on the same logic, firms with lower long/short term borrowing are likely to have lower credit ratings, because they are less likely to maintain business operations. Accrual earnings management (Dechow et al., 1995) and real earnings management (Cohen and Zarowin 2010) are established accounting measurements of managerial opportunism. We expect a negative relationship between credit ratings and managerial opportunism. Larger firms are expected to have higher credit ratings because they have more assets to maintain operations. Thus, we expect a positive relationship between firm size and credit ratings.

To control for liquidity, we include the current ratio, short-term assets divided by short term debt. Firms with more short-term assets to short term debts are less likely to default. Thus, we expect a positive relationship between liquidity and credit ratings. We estimate firm performance using return on assets (ROA). Firms with higher performance are accepted as demonstrating higher credit ratings. Next we control for ownership structures using the percentage holding of the largest domestic and foreign shareholders. Using a South Korean sample, Mali and Lim (2022) show a positive relationship exists between the percentage ownership of shareholders based on larger owners having the ability to provide guidance and break disputes between shareholders. To control for industry and year fixed-effects, we add industry dummy variables for each year and SIC codes.

2 $$\begin{aligned} {Ratings}_{i,t}=&\,{\beta }_{0}+{\beta }_{1}{TobinQ}_{i,t}+{\beta }_{2}{Lev}_{i,t}+{\beta }_{3}{SBTB}_{i,t}+{\beta }_{4}{Borrowing}_{i,t}\\ &+{\beta }_{5}{ABDA}_{i,t}+{\beta }_{6}{TRM}_{i,t}+{\beta }_{7}{Size}_{i,t}+{\beta }_{8}{Current\_R}_{i,t}\\&+{\beta }_{9}{ROA}_{i,t}+{\beta }_{10}{BigOwn}_{i,t}+{\beta }_{11}{Fore}_{i,t}+ID+YD+{\varepsilon }_{i,t} \end{aligned}$$

The Probit regression model is listed as Eq. (2). The sample selection period is from 2001 to 2016. This sample period is selected because the period excludes the effect of the 1997 Asian Financial Crisis. Moreover, following the resignation of former impeached President Park Geun-Hye, numerous business law interventions have been implemented from 2017 (Lim & Mali, 2023; You, 2021). Furthermore, from 2019, the Covid-19 pandemic has the potential to influence firm/market performance. Therefore, the 2001–2016 period can be considered a period of stability in the South Korean market. All credit rating and financial data is downloaded from the Data guide 5.0, KISVALUE, and TS-2000 (Korean) databases. The sample selection process is listed in Panel B, Table 2. Data is downloaded for all non-financial firms listed on the Korean stock market. 14,733 firm-year observations are initially downloaded. 1642 observations with insufficient financial data are then removed, leaving a final sample of 13,091 firm year observations.^1^ All our data are winsorized at bottom and top 1%.

## Empirical Results

### Univariate Analysis

In Table 3, mean, medians, maximum/minimum values, and standard deviations for each variable included in the analysis are listed. Moreover, Pearson Correlations are provided. Mean and medians are almost at parity, suggesting a normally distributed sample. Further, maximum, minimum and standard deviations are consistent with previous credit rating studies. 3.

**Table 3:** Univariate Analysis

	Obs	Mean(Med)	Max(Min)	S.D	0	1	2	3	4	5
*0. Ratings*	14,671	5.14(5)	10(1)	1.90	1					
*1. TobinQ*	13,406	0.45(0.32)	10.61(0.00)	0.48	0.25***	1				
*2. Lev*	14,733	0.43(0.43)	26.47(0.00)	0.36	− 0.46***	− 0.18***	1			
*3. Sbtb*	14,616	40.54(40.47)	99.61(0)	36.45	− 0.32***	− 0.20***	0.16***	1		
*4. Borrowing*	14,736	0.75(0.33)	4.67(0)	0.75	− 0.09***	− 0.07***	0.06***	0.03***	1	
*5. ABDA*	13,922	0.56(0.33)	0.56(0.33)	0.08	− 0.23***	0.05***	0.13***	− 0.06***	0.04***	1
*6. TRM*	13,922	− 0.03(− 0.04)	1.11(− 0.69)	0.24	− 0.16***	0.06***	0.03***	− 0.00	0.03***	0.12***
*7. Size*	13,408	24.54(24.28)	32.92(19.03)	1.55	0.25***	0.33***	− 0.11***	− 0.07***	− 0.06***	− 0.19***
*8. Current_R*	14,733	2.33(1.49)	17.73(0.24)	2.61	0.13***	0.08***	− 0.13***	− 0.13***	− 0.01	− 0.01
*9. ROA*	14,736	0.05(0.05)	1.18(− 2.75)	0.09	0.48***	0.09***	− 0.14***	− 0.06***	− 0.05***	− 0.17***
*10. BigOwn*	14,736	0.36(0.37)	1(0.00)	0.22	0.11***	− 0.17***	− 0.07***	− 0.04***	− 0.03***	− 0.12***
*11. Foreign*	14,736	0.06(0.01)	0.92(0.00)	0.11	0.23***	0.18***	− 0.09***	− 0.12***	− 0.02**	− 0.08***

	Obs	Mean(Med)	Max(Min)		6	7	8	9	10	11
*6. TRM*	13,922	− 0.03(− 0.04)	1.11(− 0.69)	0.24	1					
*7. Size*	13,408	24.54(24.28)	32.92(19.03)	1.55	− 0.00	1				
*8. Current_R*	14,733	2.33(1.49)	17.73(0.24)	2.61	0.03***	0.02*	1			
*9. ROA*	14,736	0.05(0.05)	1.18(− 2.75)	0.09	− 0.21***	0.22***	0.00	1		
*10. BigOwn*	14,736	0.36(0.37)	1(0.00)	0.22	− 0.06***	− 0.07***	0.04***	0.06***	1	
*11. Foreign*	14,736	0.06(0.01)	0.92(0.00)	0.11	0.02***	0.51***	0.03***	0.14***	0.03***	1

*, **, *** indicate significance level at 10%, 5%, 1% respectively

Refer to Table 2 for variable definitions

Pearson Correlations show the expected signs and are statistically significant at the 0.01% p-level. As inferred in hypothesis 1, Tobin’s Q increases (0.33) with credit ratings. We also find that leverage, borrowing, opportunistic earnings management and other firms risk determinants are likely to decrease credit ratings. Empirical results also show that larger firms with strong cash positions are likely to have high credit ratings.

### Multi-Variate Analysis

In Table 4, Probit regression results demonstrate the relationship between credit ratings and market performance (Tobin’s Q). After controlling for industry/year fixed effects and credit risk determinants, empirical results consistently demonstrate Tobin’s Q is increasing with credit ratings (coeff 0.30, t value 10.67). The result allows us to accept our first hypothesis. All control variables are statistically significant and demonstrate the predicted sign. All control variables are statistically significant based on our robust variable selection procedure (see Sect. 3, research design). The model can be considered robust, based on R2 and F statistic values. Based on variance inflation factor (VIF) tests, the model is shown to have no multicollinearity issues.

**Table 4:** Multi-variate analyses

Model ${Ratings}_{i,t}={\beta }_{0}+{\beta }_{1}{TobinQ}_{i,t}+{\beta }_{2}{Lev}_{i,t}+{\beta }_{3}{SBTB}_{i,t}+{\beta }_{4}{Borrowing}_{i,t}+{\beta }_{5}{ABDA}_{i,t}+{\beta }_{6}{TRM}_{i,t}+{\beta }_{7}{Size}_{i,t}+{\beta }_{8}{Current\_R}_{i,t}+{\beta }_{9}{ROA}_{i,t}+{\beta }_{10}{BigOwn}_{i,t}+{\beta }_{11}{Fore}_{i,t}+ID+YD+{\varepsilon }_{i,t}$				
	Pred	Model1	Model2	Model3
*Intercept*	+ / −	− 5.86*** (− 23.91)	− 5.71*** (− 23.36)	− 6.58*** (− 25.47)
*TobinQ*	+	0.39*** (14.74)	0.31*** (11.11)	0.30*** (10.67)
*Lev*	−	− 1.48*** (− 44.57)	− 1.47*** (− 44.36)	− 1.45*** (− 43.82)
*SBTB*	−	− 0.01*** (− 33.69)	− 0.01*** (− 34.03)	− 0.01*** (− 33.64)
*Borrowing*	−	− 0.08*** (− 19.35)	− 0.08*** (− 19.62)	− 0.08*** (− 19.47)
*ABDA*	−	− 2.38*** (− 15.90)	− 2.15*** (− 14.33)	− 2.12*** (− 14.13)
*TRM*	−	− 0.46*** (9.25)	− 0.46*** (− 9.16)	− 0.43*** (− 8.52)
*Size*	+	0.03*** (3.37)	0.03*** (3.55)	0.05*** (5.25)
*Current_R*	+	0.01*** (8.54)	0.0.1*** (8.38)	0.01*** (8.72)
*ROA*	+	7.49*** (51.73)	7.49*** (52.68)	7.42*** (51.41)
*BigOwn*	+	0.80*** (11.06)	0.50*** (8.06)	0.82*** (11.15)
*Foreign*	+	1.25*** (10.55)	1.50*** (12.86)	1.32*** (11.17)
*ID*		*Included*		*Included*
*YD*			*Included*	*Included*
*Mean VIF*		1.95	1.46	1.77
*F value*		547.98***	365.30***	285.47***
*Adj. R2*		0.5217	0.5287	0.5372
*Obs*		13,091	13,091	13,091

*, **, *** indicate significance level at 10%, 5%, 1% respectively

Refer to Table 2 for variable definitions

In Table 5, the sample is divided into two groups, firms with a Tobin’s Q of 1 < (lower that 1) and > 1 (higher than 1). Where Tobin’s Q is lower than 1, consistent with the results in Table 4, a positive relationship exists between Tobin’s Q and credit ratings (coeff 1.84, t value 27.33). This result reinforces that firms with higher market performance are expected to have higher credit ratings. However, using a sample of firms with Tobin’s Q values of higher than 1, a negative relationship exists between Tobin’s Q and credit ratings (coeff 0.16, t value −3.92). The results allow us to accept the second hypothesis.

**Table 5:** Q ratio above 1 vs Q ratio below 1

Model ${Ratings}_{i,t}={\beta }_{0}+{\beta }_{1}{TobinQ}_{i,t}+{\beta }_{2}{Lev}_{i,t}+{\beta }_{3}{SBTB}_{i,t}+{\beta }_{4}{Borrowing}_{i,t}+{\beta }_{5}{ABDA}_{i,t}+{\beta }_{6}{TRM}_{i,t}+{\beta }_{7}{Size}_{i,t}+{\beta }_{8}{Current\_R}_{i,t}+{\beta }_{9}{ROA}_{i,t}+{\beta }_{10}{BigOwn}_{i,t}+{\beta }_{11}{Fore}_{i,t}+ID+YD+{\varepsilon }_{i,t}$			
	Pred	Q ratio above 1	Q ratio below 1
*Intercept*	+ / −	− 6.23*** (− 6.52)	− 5.64*** (− 21.20)
*TobinQ*	+	− 0.16*** (− 3.92)	1.84*** (27.33)
*Lev*	−	− 1.22*** (− 9.92)	− 1.32*** (− 39.01)
*Sbtb*	−	− 0.01*** (− 8.48)	− 0.01*** (− 29.93)
*Borrowing*	−	− 0.54*** (− 9.34)	− 0.08*** (− 18.15)
*ABDA*	−	− 2.63*** (− 6.34)	− 2.25*** (− 14.43)
*TRM*	−	− 0.15 (− 1.29)	− 0.48*** (− 8.86)
*Size*	+	0.14*** (4.13)	− 0.01 (− 0.47)
*Current_R*	+	0.01*** (2.67)	0.01*** (7.98)
*ROA*	+	5.02*** (14.72)	7.49*** (47.82)
*BigOwn*	+	0.61** (2.36)	0.89*** (11.94)
*Foreign*		1.49*** (4.68)	1.21*** (9.82)
*ID*		*Included*	*Included*
*YD*		*Included*	*Included*
*Mean VIF*		3.90	1.73
*F value*		37.67***	273.45***
*Adj. R2*		0.6567	0.5479
*Obs*		1077	12,014

*, **, *** indicate significance level at 10%, 5%, 1% respectively

Refer to Table 2 for variable definitions

Empirical results suggest that after controlling for the effect of leverage and other key determinants, Tobin’s Q has an incremental effect on credit ratings. For a Q ratio below 1, the TobinQ coefficient of 1.84 means that an increase in TobinQ by 0.54 (= 1/1.84) would be a full (1–10) rating level increase. Empirical results suggest that, for example, an undervalued firm with a TobinQ of 0.90 (below 1) needs to be able to increase by 0.54 in order to achieve a one rating-level increase (a firm with Q ratio increased from 0.90 to 1.44 can increase credit rating by one). Due to the various financial benefits associated with a credit rating upgrade (Alissa et al., 2013), the results can be considered economically significant.

On the other hand, for the Q ratio above 1 sample, the TobinQ coefficient (0.16) shows that an increase in TobinQ by 6.25 (= 1/0.16) would be of a magnitude such that the rating would fall by one. It suggests that for instance, an overvalued firm with a Q ratio of 1.20 (above 1), should be able to prevent the Tobin’s Q from going up by 6.25 (too overvalued) in order to avoid experiencing one rating downgrade. Therefore, an overvalued firm with a Q ratio increase from 1.20 to 8.45 may experience a one credit rating downgrade. For completeness, we mention that in the U.S., Moody’s has 21 credit rating notches (not 10). Therefore, the results can be considered practically significant in South Korea, and internationally, if the same phenomenon exists.

The results can be more specifically interpreted as follows. Credit rating agencies may consider firms with increasing Tobin’s Q (levels of > 1) as having potentially higher credit risk, as a result of potentially being overvalued or mispriced. Therefore, increased market confidence may lead to a situation in which firms are less able to repay investor equity using assets. In short, whilst the firm performance literature infers that (Tobin’s Q is a proxy of) market performance will be associated with higher credit ratings, market confidence proxied by Tobin’s Q has the potential to become an indicator of a firm’s in-ability to survive the business cycle.

## Additional Analysis

### Incremental Analysis

**Fig. 2:**
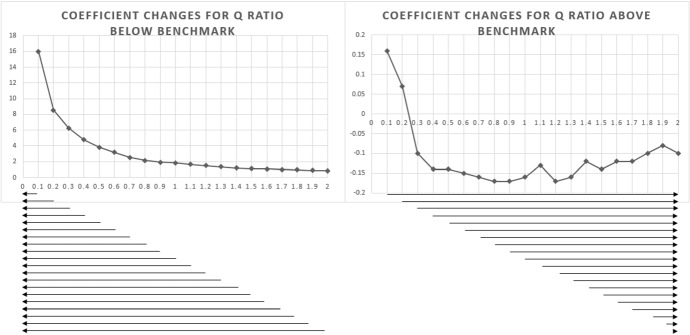
Coefficient changes for Q ratio above/below specific benchmark

In Table 6, we collect additional support for the second hypothesis, by conducting regressions for 40 different Tobin’s Q levels. The purpose of these regressions is to capture whether credit rating agencies perceive Tobin’s Q differently in relation to credit risk at specific levels. The first 20 regressions in Panel A, lists samples of firms with Tobin’s Q values of > 0.1 (all firms) to > 2.0 (only firms with the highest market confidence). Empirical results show that Tobin’s Q has a positive relationship with credit ratings for the > 0.1 (coeff 0.16, t value 6.17) and the > 0.2 (coeff 0.07, t value 2.47) samples. However, as the Tobin’s Q level increases to above 0.3 (excluding firms with Tobin’s Q of 0–0.2), the results of independent regressions suggest that the relationship changes from positive to negative (coeff −0.10, t value −3.93). From > 0.4 to 2.0, we find that Tobin’s Q has a consistent negative relationship with credit ratings, despite overall significance levels decreasing as sample size decrease. Our findings become clearer when our 20 separate regressions in Panel A are captured graphically in Fig. 2. Figure 2 clearly shows that the coefficient for firms with Tobin’s Q of > 0.2 (Obs. 9484) is statistically significantly positive, whereas the coefficient for the > 0.3 sample (Obs. 7043) is significantly negative.

**Table 6:** Sensitivity Analysis

Model ${Ratings}_{i,t}={\beta }_{0}+{\beta }_{1}{TobinQ}_{i,t}+{\beta }_{2}{Lev}_{i,t}+{\beta }_{3}{SBTB}_{i,t}+{\beta }_{4}{Borrowing}_{i,t}+{\beta }_{5}{ABDA}_{i,t}+{\beta }_{6}{TRM}_{i,t}+{\beta }_{7}{Size}_{i,t}+{\beta }_{8}{Current\_R}_{i,t}+{\beta }_{9}{ROA}_{i,t}+{\beta }_{10}{BigOwn}_{i,t}+{\beta }_{11}{Fore}_{i,t}+ID+YD+{\varepsilon }_{i,t}$										
*Panel A: Q ratio above specific benchmark*										
	Q > 0.1	Q > 0.2	Q > 0.3	Q > 0.4	Q > 0.5	Q > 0.6	Q > 0.7	Q > 0.8	Q > 0.9	Q > 1.0
*Intercept*	− 5.92*** (− 22.65)	− 5.49*** (− 18.32)	− 5.62*** (− 17.94)	− 5.53*** (− 14.61)	− 5.38*** (− 11.76)	− 5.91*** (− 11.03)	− 6.12*** (− 9.96)	− 6.10*** (− 8.48)	− 6.28*** (− 7.53)	− 6.23*** (− 6.52)
*TobinQ*	0.16*** (6.17)	0.07** (2.47)	− 0.10*** (− 3.93)	− 0.14*** (− 4.95)	− 0.14*** (− 4.65)	− 0.15*** (− 4.48)	− 0.16*** (− 4.56)	− 0.17*** (− 4.59)	− 0.17*** (− 4.37)	− 0.16*** (− 3.92)
*Controls*	*Included*	*Included*	*Included*	*Included*	*Included*	*Included*	*Included*	*Included*	*Included*	*Included*
*ID/YD*	*Included*	*Included*	*Included*	*Included*	*Included*	*Included*	*Included*	*Included*	*Included*	*Included*
*Mean VIF*	1.90	2.07	2.29	2.59	2.70	2.89	3.04	3.23	3.42	3.90
*F value*	286.45***	212.65***	235.32***	164.63***	115.51***	85.77***	64.65***	51.79***	41.72***	37.67***
*Adj. R2*	0.5610	0.5445	0.6409	0.6347	0.6263	0.6237	0.6252	0.6298	0.6299	0.6567
*Obs*	11,936	9484	7043	5075	3707	2797	2108	1636	1328	1077
	Q > 1.1	Q > 1.2	Q > 1.3	Q > 1.4	Q > 1.5	Q > 1.6	Q > 1.7	Q > 1.8	Q > 1.9	Q > 2.0
*Intercept*	− 6.48*** (− 5.77)	− 7.76*** (− 6.11)	− 8.57*** (− 5.59)	− 6.38*** (− 3.29)	− 10.68*** (− 6.46)	− 10.33*** (− 5.53)	− 10.15*** (− 4.75)	− 10.53*** (− 4.50)	− 10.99*** (− 4.29)	− 10.39*** (− 3.89)
*TobinQ*	− 0.13*** (− 3.08)	− 0.17*** (− 3.65)	− 0.16*** (− 3.27)	− 0.12*** (− 2.34)	− 0.14*** (− 2.98)	− 0.12** (− 2.36)	− 0.12** (− 2.34)	− 0.10* (− 1.93)	− 0.08 (− 1.47)	− 0.10* (− 1.70)
*Controls*	*Included*	*Included*	*Included*	*Included*	*Included*	*Included*	*Included*	*Included*	*Included*	*Included*
*ID/YD*	*Included*	*Included*	*Included*	*Included*	*Included*	*Included*	*Included*	*Included*	*Included*	*Included*
*Mean VIF*	4.88	4.99	5.84	13.04	6.64	6.68	10.37	9.53	8.43	8.08
*F value*	30.03***	24.61***	19.92***	17.82***	24.56***	26.35***	25.44***	21.22***	17.48***	17.17
*Adj. R2*	0.6516	0.6561	0.6590	0.6665	0.7735	0.8098	0.8291	0.8261	0.8301	0.8396
*Obs*	888	724	589	477	386	325	282	247	207	185
*Panel B: Q ratio below specific benchmark*										
	Q < 0.1	Q < 0.2	Q < 0.3	Q < 0.4	Q < 0.5	Q < 0.6	Q < 0.7	Q < 0.8	Q < 0.9	Q < 0.10
*Intercept*	− 6.57*** (− 8.36)	− 6.21*** (− 13.47)	− 5.58*** (− 15.24)	− 5.39*** (− 16.65)	− 5.49*** (− 18.31)	− 5.32*** (− 18.55)	− 5.37*** (− 19.30)	− 5.45*** (− 20.08)	− 5.48*** (− 20.54)	− 5.64*** (− 21.20)
*TobinQ*	16.01*** (9.83)	8.54*** (17.96)	6.26*** (24.69)	4.78*** (28.51)	3.82*** (29.71)	3.17*** (29.88)	2.55*** (28.26)	2.16*** (27.19)	1.92*** (26.48)	1.83*** (27.33)
*Controls*	*Included*	*Included*	*Included*	*Included*	*Included*	*Included*	*Included*	*Included*	*Included*	*Included*
*ID/YD*	*Included*	*Included*	*Included*	*Included*	*Included*	*Included*	*Included*	*Included*	*Included*	*Included*
*Mean VIF*	1.44	1.45	1.53	1.59	1.64	1.67	1.69	1.71	1.72	1.73
*F value*	22.86***	68.61***	118.31***	164.17***	204.92***	231.76***	251.00***	266.35***	276.12***	273.45***
*Adj. R2*	0.5087	0.5058	0.5113	0.5222	0.5379	0.5454	0.5490	0.5532	0.5555	0.5479
*Obs*	1155	3607	6048	8016	9384	10,294	10,983	11,455	11,763	12,014
	Q < 1.1	Q < 1.2	Q < 1.3	Q < 1.4	Q < 1.5	Q < 1.6	Q < 1.7	Q < 1.8	Q < 1.9	Q < 2.0
*Intercept*	− 5.70*** (− 21.61)	− 5.75*** (− 21.91)	− 5.80*** (− 22.22)	− 5.85*** (− 22.50)	− 5.87*** (− 22.62)	− 5.88*** (− 22.70)	− 5.93*** (− 22.88)	− 5.96*** (− 23.03)	− 6.01*** (− 23.23)	− 6.04*** (− 23.34)
*TobinQ*	1.66*** (26.54)	1.50*** (25.56)	1.34*** (24.26)	1.22*** (23.43)	1.14*** (22.98)	1.09*** (22.78)	1.03*** (22.12)	0.98*** (21.61)	0.91*** (20.51)	0.87*** (19.97)
*Controls*	*Included*	*Included*	*Included*	*Included*	*Included*	*Included*	*Included*	*Included*	*Included*	*Included*
*ID/YD*	*Included*	*Included*	*Included*	*Included*	*Included*	*Included*	*Included*	*Included*	*Included*	*Included*
*Mean VIF*	1.73	1.74	1.75	1.75	1.75	1.76	1.76	1.76	1.76	1.76
*F value*	280.42***	285.40***	287.28***	290.28***	289.49***	289.56***	289.53***	290.59***	290.55***	290.69***
*Adj. R2*	0.5502	0.5513	0.5502	0.5505	0.5481	0.5470	0.5461	0.5463	0.5455	0.5452
*Obs*	12,203	12,367	12,502	12,614	12,705	12,766	12,809	12,844	12,884	12,906

*, **, *** indicate significance level at 10%, 5%, 1% respectively

Refer to Table 2 for variable definitions

The result in Table 6, Panel B demonstrate consistent results when we include individual regressions for firms with Tobin’s Q values of < 0.1 (only firms with a Tobin’s Q of 0.1) to < 2.0 (all firms). We find a strong positive coefficient (16.01) for firms with Tobin’s Q values of below 0.1. The relationship becomes less positive at 0.2 (coeff, 8.54). As the Tobin’s Q values increases, coefficients also decrease. As shown in Fig. 2 and Panel B, the positive association continues to become less significantly positive suggesting that the positive association between credit ratings and Tobin’s Q is predominantly determined by firms with relatively low Tobin’s Q levels.

The findings have important implications. In the first analysis (hypothesis), we demonstrate a positive relationship between credit ratings and Q ratios. In the second analysis, we show that firms with a Tobin’s Q of 1 < are more likely to have a positive credit rating as Tobin’s Q increases. However, firms with a Tobin’s Q value of > 1 are more likely to have a negative relation. In the incremental analysis, we demonstrate that, (i) the positive relationship between a credit rating analyst's perception of risk and Tobin’s Q is likely based on firms with relatively low Tobin’s Q levels (0.3 <). Firms with a Tobin’s Q of below 0–0.2 can be considered as having relatively low market confidence; thus, higher market confidence (Tobin’s Q) for such firms can reduce credit risk. (ii) We demonstrate that holding all firm risk variables consistent, a threshold level can be captured (> 0.3) at which an incrementally higher Q ratio has a negative influence credit rating. The results also show that market performance does not indicate a firm's ability to survive the business cycle per se*,* and that credit rating analysts likely utilize specific vectors-based Tobin’s Q level models using AI technology and machine learning to capture the effect of market confidence on credit ratings at specific levels.

### Persistency Tests

To add robustness, we determine whether Tobin’s Q can have a positive effect on credit ratings in subsequent periods. Therefore, in Table 7, we capture the influence of Tobin’s Q on credit ratings in period t + 1 and period t + 2. We believe it is important to capture the relationship between Tobin’s Q and credit ratings in subsequent periods because previous studies demonstrate that credit rating agencies have an incentive to keep credit ratings consistent. Becker and Milbourn (2011) suggest that credit ratings analysts are unlikely to modify a credit rating without sufficient evidence and therefore take a long-term approach. Hovakimian and Hovakimian (2009) surmise that credit ratings are designed to be a forward-looking estimate of credit risk. Altman and Rijken (2004) explain that credit ratings are only updated only when an analyst is confident that changes in a firm's default risk position is permanent, an organizational phenomenon known as the prudent rating migration policy. We find that Tobin’s Q has a positive influence on credit ratings in period t + 1 (coeff 0.36, t value 10.45) and t + 2 (coeff 0.32, t value 8.13). The result suggests that Tobin’s Q influence credit ratings in both the current and subsequent periods, consistent with credit rating analysts interpreting Q ratios based on a long-term approach.

**Table 7:** Persistency tests

Model ${Ratings}_{i,t(t+1,t+2)}={\beta }_{0}+{\beta }_{1}{TobinQ}_{i,t}+{\beta }_{2}{Lev}_{i,t}+{\beta }_{3}{SBTB}_{i,t}+{\beta }_{4}{Borrowing}_{i,t}+{\beta }_{5}{ABDA}_{i,t}+{\beta }_{6}{TRM}_{i,t}+{\beta }_{7}{Size}_{i,t}+{\beta }_{8}{Current\_R}_{i,t}+{\beta }_{9}{ROA}_{i,t}+{\beta }_{10}{BigOwn}_{i,t}+{\beta }_{11}{Fore}_{i,t}+ID+YD+{\varepsilon }_{i,t}$				
	Pred	DV:${Ratings}_{i,t}$	DV:${Ratings}_{i,t+1}$	DV:${Ratings}_{i,t+2}$
*Intercept*	+ / −	− 6.58*** (− 25.47)	− 6.01*** (− 20.32)	− 5.90*** (− 17.71)
*TobinQ*	+	0.30*** (10.67)	0.36*** (10.45)	0.32*** (8.13)
*Lev*	−	− 1.45*** (− 43.82)	− 1.15*** (− 31.27)	− 0.93*** (− 23.01)
*SBTB*	−	− 0.01*** (− 33.64)	− 0.01*** (− 25.55)	− 0.01*** (− 21.68)
*Borrowing*	−	− 0.08*** (− 19.47)	− 0.07** (− 13.61)	− 0.05*** (− 9.00)
*ABDA*	−	− 2.12*** (− 14.13)	− 2.01*** (− 11.72)	− 1.77*** (− 9.25)
*TRM*	−	− 0.43*** (− 8.52)	− 0.58*** (− 10.13)	− 0.45*** (− 6.96)
*Size*	+	0.05*** (5.25)	0.02** (2.08)	0.03** (2.01)
*Current_R*	+	0.01*** (8.72)	0.01*** (6.75)	0.01*** (6.64)
*ROA*	+	7.42*** (51.41)	6.57*** (40.18)	5.35*** (29.56)
*BigOwn*	+	0.82*** (11.15)	0.83*** (10.04)	0.83*** (8.94)
*Foreign*	+	1.32*** (11.17)	1.63*** (12.08)	1.72*** (11.27)
*ID*		*Included*	*Included*	*Included*
*YD*		*Included*	*Included*	*Included*
*Mean VIF*		1.77	1.73	1.71
*F value*		285.47***	180.88***	114.06***
*Adj. R2*		0.5372	0.4368	0.3413
*Obs*		13,091	12,182	11,278

*, **, *** indicate significance level at 10%, 5%, 1% respectively

Refer to Table 2 for variable definitions

### Controlling for Previous Year’s Credit Ratings

A firm’s credit rating in the previous year can also be considered a determinant for subsequent credit ratings (Avramov et al., 2007). Therefore, to add robustness, previous year’s credit ratings are included in Table 8. Results are consistent when a firm’s previous credit rating is included into the model using the t-1 (coeff 0.08, t value 3.84) and differenced approach (coeff 0.25, t value 9.36). The results provide further evidence 1of model robustness.

**Table 8:** Controlling for previous ratings/rating changes

Model ${Ratings}_{i,t}={\beta }_{0}+{\beta }_{1}{TobinQ}_{i,t}+{\beta }_{2}{Lev}_{i,t}+{\beta }_{3}{SBTB}_{i,t}+{\beta }_{4}{Borrowing}_{i,t}+{\beta }_{5}{ABDA}_{i,t}+{\beta }_{6}{TRM}_{i,t}+{\beta }_{7}{Size}_{i,t}+{\beta }_{8}{Current\_R}_{i,t}+{\beta }_{9}{ROA}_{i,t}+{\beta }_{10}{BigOwn}_{i,t}+{\beta }_{11}{Fore}_{i,t}+{\beta }_{12}{{Ratings}_{i,t-1}/\Delta Ratings}_{i,t}+ID+YD+{\varepsilon }_{i,t}$			
	Pred	DV: CR_t	DV: CR_t
*Intercept*	+ / −	− 1.17*** (− 6.21)	− 6.71*** (− 27.13)
*TobinQ*	+	0.08*** (3.84)	0.25*** (9.36)
*Lev*	−	− 0.85*** (− 28.90)	− 1.88*** (− 50.58)
*SBTB*	−	− 0.00*** (− 18.69)	− 0.01*** (− 32.23)
*Borrowing*	−	− 0.04*** (− 12.30)	− 0.08*** (− 17.80)
*ABDA*	−	− 1.21*** (− 11.11)	− 1.87*** (− 12.86)
*TRM*	−	− 0.22*** (− 6.20)	− 0.41*** (− 8.36)
*Size*	+	− 0.02*** (− 2.81)	0.07*** (7.29)
*Current_R*	+	0.00*** (3.33)	0.01*** (7.96)
*ROA*	+	4.73*** (43.41)	6.70*** (46.27)
*BigOwn*	+	0.06 (1.03)	0.86*** (11.97)
*Foreign*	+	0.42*** (5.06)	1.28*** (11.41)
${Ratings}_{i,t-1}$		0.61*** (107.56)	
${\Delta Ratings}_{i,t}$			0.31*** (29.99)
*ID*		*Included*	*Included*
*YD*		*Included*	*Included*
*Mean VIF*		1.70	1.68
*F value*		803.60***	342.71***
*Adj. R2*		0.7732	0.5925
*Obs*		12,544	12,544

*, **, *** indicate significance level at 10%, 5%, 1% respectively

Refer to Table 2 for variable definitions

***${Ratings}_{i,t-1}$ and ${\Delta Ratings}_{i,t}$ are credit ratings in previous year and ratings changes respectively

### Investment/Non-Investment Grade Analysis

Next, we estimate whether the relationship between credit ratings and Tobin’s Q is consistent for IG and NIG firms. The literature suggests that financial institutions, regulators, market participants and accounting professionals consider firms above the IG threshold as having lower levels of default risk, compared to NIG firms (Alissa et al., 2013; Becker & Milbourn, 2011; Bolton et al., 2012; Kraft, 2015; Opp et al., 2013). Alissa et al. (2013) suggests that firms that are below the IG threshold have explicit incentives to belong to the IG group, because IG firms enjoy better terms from various groups including suppliers, banks, and financial institutions. Mali and Lim (2019) also demonstrate that firms above the IG threshold are considered differently based on efficiency performance. Therefore, we question whether credit rating agencies perceive Tobin’s Q performance differently for both groups.

In Table 9, Panel A, a positive relation is demonstrated between Tobin’s Q and credit ratings for NIG (coeff 0.23, t value 6.24). However, for the IG sample a negative relationship exists (coeff −0.11, t value −5.67). Because firms with low credit ratings are expected to have higher risk and lower market confidence, a small increase in Tobin’s Q can have a large positive effect on credit ratings. The IG group analysis can be interpreted as follows. For firms with very low (high) credit risk (ratings), incrementally increasing levels of Tobin’s Q may be a signal of increasing investment momentum, which is likely to be perceived by rating agencies as increasing default risk, due to firm overvaluation/mispricing, in an instance where investors would be inclined to sell shares.

**Table 9:** Comparative Analysis

Model ${Ratings}_{i,t}={\beta }_{0}+{\beta }_{1}{TobinQ}_{i,t}+{\beta }_{2}{Lev}_{i,t}+{\beta }_{3}{SBTB}_{i,t}+{\beta }_{4}{Borrowing}_{i,t}+{\beta }_{5}{ABDA}_{i,t}+{\beta }_{6}{TRM}_{i,t}+{\beta }_{7}{Size}_{i,t}+{\beta }_{8}{Current\_R}_{i,t}+{\beta }_{9}{ROA}_{i,t}+{\beta }_{10}{BigOwn}_{i,t}+{\beta }_{11}{Fore}_{i,t}+ID+YD+{\varepsilon }_{i,t}$			
Panel A: Investment grade vs Non − investment grade group			
	Pred	IG	NIG
*Intercept*	+ /–	− 5.20*** (− 24.53)	− 7.56*** (− 26.60)
*TobinQ*	+	− 0.11*** (− 5.67)	0.23*** (6.24)
*Lev*	–	− 3.77*** (− 40.49)	− 0.44*** (− 17.09)
*Sbtb*	–	− 0.00*** (− 11.04)	− 0.00*** (− 6.13)
*Borrowing*	–	− 0.20*** (− 4.47)	− 0.05*** (− 15.06)
*ABDA*	–	− 1.57*** (11.36)	− 1.20*** (− 8.27)
*TRM*	–	− 0.22*** (− 5.58)	− 0.30*** (− 5.04)
*Size*	+	0.07*** (9.36)	0.03*** (2.87)
*Current_R*	+	− 0.00** (− 2.44)	0.04*** (8.64)
*ROA*	+	5.34*** (37.91)	3.26*** (21.15)
*BigOwn*	+	0.33*** (5.35)	0.24*** (3.10)
*Foreign*	+	0.44*** (5.24)	0.51*** (2.90)
*ID*		*Included*	*Included*
*YD*		*Included*	*Included*
*Mean VIF*		2.00	1.62
*F value*		142.75***	40.02***
*Adj. R2*		0.5004	0.2809
*Obs*		7607	5484

*, **, *** indicate significance level at 10%, 5%, 1% respectively

Refer to Table 2 for variable definitions

### OLS/Controlling for Firm/Year Fixed Effect

In the main analysis, Probit regression analysis is conducted, using dummy variables to control for industry/year fixed effects. To reduce the potential of time series/industry dependence, we re-perform additional analyses including the (i) Fama–MacBeth (1973) fixed effect technique and (ii) clustering for standard errors at firm level. Untabulated results show consistent results for all analyses. Furthermore, as an additional robustness check, we re-run all above analyses using OLS regression. As expected, OLS regression and Probit regression analysis results are qualitatively indifferent.

### Excluding the Leverage Effect

In the main analysis, we examine the relationship between credit ratings and Tobin’s Q, after controlling for key credit rating determinants. Leverage is included as a control variable, because it is a key credit rating determinant. However, there is the potential that multicollinearity exists between ${\beta }_{1}$ TobinQ and ${\beta }_{2}$ leverage, since TobinQ estimation includes liability size. The Pearson correlation between TobinQ and Lev is − 0.18 (−18%). However, mean VIF is far below 10, suggesting that the model is free from multicollinearity problems. Regardless, for robustness, in order to rule out the ‘leverage effect’, the main analysis is repeated after excluding leverage. Untabulated results show that in the absence of leverage, empirical findings are consistent with the main analysis (TobinQ Coeffi −0.19 t value −2.22 for Q ratio above 1, Coeffi 2.11 t value 33.54 for Q ratio below 1).

### Leverage Above/Below Median Analysis

In Sect. 5.6, we exclude the effect of leverage and repeat the main analyses. However, the negative effect of the coefficient on TobinQ may be caused by a group of firms with significantly high TobinQ. It is also possible that given that the definition of TobinQ includes the ratio of total liabilities to assets, firms with high TobinQ may include those with high liabilities. In order to clarify the issue, we repeat the main analyses after dividing the sample into two groups: (1) firms with leverage above median, and (2) below median.

In Table 10, a positive relation is shown between TobinQ and credit ratings for the leverage above median sample (coeff 0.49, t value 12.20). However, for the leverage below median sample, a negative relationship is demonstrated (coeff −0.06, t value −2.56). Because firms with high leverage are expected to have higher risk and lower market confidence, a small increase in TobinQ can have a large positive effect on credit ratings. The leverage below median analysis can be interpreted as follows. For firms with very low (high) credit risk (ratings), incrementally increasing levels of TobinQ may be a signal of increasing investment momentum, which is likely to be perceived by rating agencies as increasing default risk, due to firm overvaluation/mispricing, in an instance where investors would be inclined to sell shares.

**Table 10:** Leverage above median vs Leverage below median

Model ${Ratings}_{i,t}={\beta }_{0}+{\beta }_{1}{TobinQ}_{i,t}+{\beta }_{2}{Lev}_{i,t}+{\beta }_{3}{SBTB}_{i,t}+{\beta }_{4}{Borrowing}_{i,t}+{\beta }_{5}{ABDA}_{i,t}+{\beta }_{6}{TRM}_{i,t}+{\beta }_{7}{Size}_{i,t}+{\beta }_{8}{Current\_R}_{i,t}+{\beta }_{9}{ROA}_{i,t}+{\beta }_{10}{BigOwn}_{i,t}+{\beta }_{11}{Fore}_{i,t}+ID+YD+{\varepsilon }_{i,t}$			
	Pred	Lev above median	Lev below median
*Intercept*	+ / −	6.05***	6.41***
		(25.95)	(36.05)
*TobinQ*	+	0.49***	− 0.06**
		(12.20)	(2.56)
*Lev*		− 0.48***	− 3.26***
		(− 14.15)	(− 23.11)
*Sbtb*	–	− 0.01***	− 0.00***
		(− 13.36)	(− 2.74)
*Borrowing*	–	− 0.05***	− 2.56***
		(− 12.82)	(− 26.10)
*ABDA*	–	− 2.07***	− 2.28***
		(− 11.16)	(− 14.70)
*TRM*	–	− 0.69***	− 0.24***
		(− 11.44)	(− 5.89)
*Size*	+	− 0.07***	0.08***
		(− 5.94)	(8.46)
*Current_R*	+	0.08***	− 0.00***
		(7.60)	(− 3.27)
*ROA*	+	7.29***	7.39***
		(39.91)	(53.96)
*BigOwn*	+	0.34***	0.31***
		(4.21)	(5.21)
*Foreign*	+	2.38***	0.46***
		(13.31)	(4.65)
*ID*		*Included*	*Included*
*YD*		*Included*	*Included*
*Mean VIF*		1.13	1.47
*F value*		403.52***	959.20***
*Adj. R2*		0.4044	0.6177
*Obs*		6550	6541

*, **, *** indicate significance level at 10%, 5%, 1% respectively

Refer to Table 2 for variable definitions

## Conclusion and Discussion

This study is important for numerous reasons. First, based on previous studies that associate credit rating and firm performance, Tobin’s Q (market performance) can be expected to have a positive association with credit ratings. Using Probit/OLS regression for a sample of South Korean listed firms, a positive relationship exists between Tobin’s Q and credit ratings. On face value, the results suggest that firms with higher market performance are more likely to survive the business cycle. However, when the sample is divided into firms with a Tobin’s Q of 1 < and > 1, market performance does not have a linear relationship with credit ratings. Firms with a Tobin’s Q of 1 < continue to demonstrate a positive relation, whilst firms with a Tobin’s Q of > 1 demonstrate a negative relation. Firms with a Q ratio of 1 < are able to repay investors’ equity share using assets, if investors were inclined to sell their equity holding. Firms with a > 1 are not. Therefore, firms with a Q ratio of > 1 are in a riskier position if investors decided to sell their equity share. The default risk assertion of rating’s analysts is likely to be confounded as a result of potential overvaluation/mispricing. The study therefore adds granularity to Tobin’s Q as a signal of default risk, and a firm performance proxy. The study also provides evidence that credit rating agencies are nuanced when making default risk assertions, based on Tobin’s Q status.

Second, to add robustness to the aforementioned finding, additional analysis is conducted for firms with multiple Tobin’s Q levels. A positive relationship exists between Tobin’s Q and credit rating for firms with a Tobin’s Q of 0.2 < . However, at a Q ratio of above 0.3, the relationship between market Tobin’s Q and credit ratings (risk) is negative (positive). The results suggest that for firms with relatively low market confidence, but achieve a 100% asset to equity value increase (0.075–0.15), the effect on credit ratings is highly positive. The results also suggest that a threshold level exists, based on Tobin’s Q, at which the positive association between credit ratings and Tobin’s Q becomes negative. To the best of our knowledge, this is the first study to report that a ‘threshold level’ exists. To generalize this study, we encourage future studies to capture whether or not a similar threshold level exists in other economies. Third, the study provides empirical evidence that in South Korea, the association between Tobin’s Q and credit ratings is negative (positive) for NIG (IG) firms. The result suggest that for NIG firms with lower market value and higher credit risk, investor confidence has a significant positive effect. On the other hand, for low-risk, high market value firm IG, the relationship is negative. The results therefore provide further evidence that firm characteristics, based on Tobin’s Q, are included in rating’s analysts default risk assessments.

Fourth, weak financial legislation is shown to be a contributing factor in the 1997 Korean (Asian) Financial Crisis (Emst, 1998; LaPorta, 1997). Following the crisis, South Korean legislators have implemented policies to enhance financial reporting quality (Lim & Mali, 2020; Mali & Lim, 2018, 2019, 2020). The study therefore provides academics with a basis to evaluate whether instances of national crises can influence a rating agency’s perception about Tobin’s Q and credit ratings. There is the potential that the positive association between Tobin’s Q and credit ratings, becomes negative, in South Korea, because of market overconfidence and earnings management, associated with the Financial Crisis. The 0.2/0.3 threshold level in South Korea is lower than expected. We conjecture that in established economies, it will be higher. We strongly recommend that comparative ‘threshold level’ analyses investigate whether the credit rating/Tobin’s Q threshold level exists in the U.S. and UK firms, and whether it is higher compared to South Korean. Evidence of the aforementioned has the potential to offer insights to market participants about the credit risk associated with investor confidence on an international basis, based on firm-level data.

Fifth, in accounting and finance classrooms, Tobin’s Q is contextualized as a proxy for market performance, with increasing market performance being a signal of investor confidence. This study provides evidence that Tobin’s Q as a market performance indicator should come with a caveat. More specifically, as Tobin’s Q values become increasingly high based on investor confidence, credit rating agencies perceive that a firm is less likely to survive a business cycle.

Finally, limitations are listed. First, the study uses the credit ratings of only one credit rating agency (NICE). We are unable to utilize the data of other South Korean credit rating agencies because of limited data unavailability. We encourage future studies to repeat this analysis using a comparative analysis approach that includes the credit ratings of various agencies. The result of such a study could be developed into a rating shopping analysis to capture whether rating agencies accommodate clients.

Second, the Tobin’s Q measure is strongly influenced by market capitalization since the numerator of TQ includes the market value of common and preferred stock. Thus, pricing behavior in the Korean stock market may influence the results. According to Kim et al. (2022), the Korean market has been boxed during 2012–2016. They also mention that active investors rather than passive investors dominate the Korean stock market. Therefore, in this period, there may be a possibility that stock price deviations from fundamental levels may affect the results.

Third, univariate analysis (Table 3) shows that the distribution of TQ appears to be skewed. Although we winzorized data at the bottom and top 1% levels to control for the outlier effect, the mean is 0.45, and the median is 0.32. Thus, firms with high TQ may pull the distribution to the right. The standard deviation is also significant at 0.48, therefore, the distribution of firm size follows a fat-tailed distribution (Gabaix, 2011), inferring that the behavior of some very large firms such as Samsung electronics may have a significant impact on aggregate market trends. We encourage future studies to repeat the analysis using international data to discover whether the results are a universal phenomenon or only observed in South Korea.

## Data Availability

Data will be deposited in the University of Nottingham repository upon publication. All persons who meet authorship criteria are listed as authors, and all authors certify that they have participated sufficiently in the work to take public responsibility for the content, including participation in the concept, design, analysis, writing, or revision of the manuscript. Furthermore, each author certifies that this material or similar material has not been and will not be submitted to or published in any other publication.

## Footnotes

1 The data that support the findings of this study are openly available in figshare at https://figshare.com/articles/Tobin’sQ_xlsx/11947959.

## Declarations

### Human and Animal participants

The research does not involve human participants.
